# Dosimetric comparison between RapidArc and HyperArc techniques in salvage stereotactic body radiation therapy for recurrent nasopharyngeal carcinoma

**DOI:** 10.1186/s13014-020-01602-7

**Published:** 2020-07-08

**Authors:** Hsiu-Wen Ho, Steve P. Lee, Hisu-Man Lin, Hsiao-Yun Chen, Chun-Chiao Huang, Shih-Chang Wang, Ching-Chieh Yang, Yu-Wei Lin

**Affiliations:** 1 Department of Radiation Oncology, Chi Mei Medical Center, No.901, Jhonghua Rd., Yongkang Dist., Tainan City, 71004 Taiwan; 2 Department of Radiation Oncology, David Geffen School of Medicine, University of California, Los Angeles, CA USA; 3 Department of Pharmacy, Chia-Nan University of Pharmacy and Science, Tainan, Taiwan

**Keywords:** Recurrent nasopharyngeal carcinoma, RapidArc, HyperArc, Stereotactic body radiation therapy (SBRT), Dosimetric comparison, Biologically effective dose (BED)

## Abstract

**Background:**

To evaluate dosimetric differences of salvage irradiations using two commercially available volumetric modulated arc therapy (VMAT) stereotactic body radiation therapy (SBRT) techniques: RapidArc (RA) and HyperArc (HA), for recurrent nasopharyngeal carcinoma (NPC) after initial radiation therapy.

**Methods:**

Ten patients with recurrent NPC status previously treated with radiation therapy were considered suitable candidates for salvage SBRT using VMAT approach. Two separate treatment plans were created with HA and RA techniques for each case, with dosimetric outcomes compared with respect to tumor target coverage and organs-at-risk (OARs) sparing. Furthermore, the cumulative radiobiological effects to the relevant OARs from the original radiotherapy to the respective salvage SBRT plans were analyzed in terms of biologically effective dose (BED).

**Results:**

Treatment with HA exhibited similar target dose coverage as with RA, while delivering a higher mean dose to the targets. Using RA technique, the mean maximal doses to optic apparatus and the mean brain dose were reduced by 1 to 1.5 Gy, comparing to HA technique. The conformity index, gradient radius, and intermediate dose spillage in HA plans were significantly better than those in RA. With HA technique, the volume of brain receiving 12 Gy or more was reduced by 44%, comparing to RA technique. The cumulative BEDs to spinal cord and optic apparatus with RA technique were 1 to 2 Gy_3_ less than those with HA. HA technique significantly reduced the volume within body that received more than 100 Gy.

**Conclusions:**

With better dose distribution than RA while maintaining sufficient target dose coverage, HA represents an attractive salvage SBRT technique for recurrent NPC.

## Background

Radiation therapy with or without concurrent chemotherapy represents the standard treatment for nasopharyngeal cancer (NPC) and leads to a 5-year local control rate of greater than 85% [1–9]. However, local recurrence still represents a major source of morbidity and mortality in patients with advanced stage NPC [10, 11]. Because of the invasive nature of the malignancy which may involve many critical tissues at skull base, radiation therapy often remains to be the main salvage treatment modality once the disease recurs locally.

The main feature of stereotactic body radiation therapy (SBRT) is the delivery of relatively few fractions of ultra-high dose coverage tightly conformed to the intended target volumes, while minimizing dosages to adjacent critical organs at risk (OARs). Many studies have documented that SBRT may improve tumor control, reduce toxicity, and improve quality of life in patients with recurrent head and neck cancer, including NPC [12–16].

RapidArc (RA, Varian Medical System, Palo Alto, CA, USA) is an isocentric-coplanar volumetric-modulated arc radiotherapy (VMAT) technique that can deliver highly conformal, intensity-modulated radiation therapy (IMRT) doses via a single arc or multiple rotations of the gantry of a linear accelerator [17]. RA enables treatment plans with an improved dosimetric outcome as compared to multifield IMRT while reducing the treatment time per fraction in the SBRT setting [18].

HyperArc (HA, Varian Medical System) is a relatively new isocentric VMAT technique developed specifically for non-coplanar, multileaf collimator (MLC)-based stereotactic radiotherapy with automated treatment optimization and dose delivery [19, 20]. HA has been demonstrated as a novel stereotactic radiosurgery (SRS) technique for single or multiple brain metastasis [21–23]. To our best knowledge, however, few studies have explored the ability of HA to generate high-quality treatment plans for extracranial lesions – especially as pertains to SBRT which is gaining popularity as a salvage measure for recurrent head & neck malignancies. In such case, since SBRT for re-irradiation often utilizes few fractions of relatively high dosage while the initial radiotherapy mainly follows conventional fractionation scheme, the combined biologic or clinical effect at any anatomic site of interest (i.e. critical OARs) cannot be inferred from the simple summation of physical dosages received in the sequentially separate treatment courses. Rather, corrections using the concept of biologically effective dose (BED) or equivalent dose to 2-Gy per fraction (EQD2) may be used in order to analyze the ultimate dosimetric consequences. This would be of crucial importance when offering curative SBRT to re-irradiate recurrent NPC where the anatomic region is filled with critical normal structures. Upon inverse planning for SBRT, the “dose” constraints in the conventional dose-volume histogram (DVH) should likewise be converted to BED in order to facilitate meaningful comparisons for dosimetric outcome evaluations.

The aim of the current study was to evaluate the dosimetric differences between the RA and HA SBRT techniques for the salvage treatment of recurrent NPC after initial primary radiation therapy, factoring radiobiologically-corrected cumulative doses to critical OARs as part of DVH objective parameters upon IMRT inverse planning.

## Methods

### Study groups

Ten patients with recurrent NPC who had been treated with initial primary radiation therapy and subsequent salvage SBRT were enrolled. Their clinical and dosimetric characteristics are shown in Table 1. The RA and HA treatment plans were created retrospectively for each patient to meet a previously set and radiobiologically sound salvage SBRT planning criteria (Table 2).

**Table 1:** Characteristics of the recurrent nasopharyngeal cancer patients

Parameters	
Patient number	10
Primary radiation therapy (Gy/fractions)	
Median	70/35
Range	64/32–70/35
Recurrent T stage	
T1	1
T2	4
T3	1
T4	4
CTV (cm^3^)	
Median	14.9
Range	1.5–37.1
PTV (cm^3^)	
Median	17.2
Range	2.4–51.8
Salvage SBRT dose (Gy)	
Median	36.8
Range	32.5–40.0
Fractions	5

Abbreviations: *CTV* Clinical target volume, *PTV* Planning target volume

**Table 2:** Planning objectives for the target and organs at risk

Objectives	Parameters/organs	Tolerance	Priority
Target	Maximum dose	≤ 120% of the prescription dose	2
	Coverage (minimal)	95% of the prescription dose cover 95% of the PTV	2
Organs at risk	Spinal cord	Dmax < 10 Gy or Cumulative dose of EQD2 < 50Gy (83.33Gy_3_)	1
	Brainstem	Dmax < 13 Gy or Cumulative dose of EQD2 < 54Gy (90.00Gy_3_)	1
	Optic nerve	Dmax < 12 Gy or Cumulative dose of EQD2 < 50Gy (83.33Gy_3_)	2
	Chiasm	Dmax <15Gy or Cumulative dose of EQD2 < 54Gy (90.00Gy_3_)	1
	Eye	Dmax < 10 Gy or Cumulative dose of EQD2 < 50Gy (83.33Gy_3_)	2
	Lens	Dmax <4Gy	3

Abbreviations: *Dmax* The maximal point dose of the organ at risk *EQD2* Equivalent dose to 2 Gy per fraction, *Gy*_*3*_ Unit of BED with α/β ratio of 3 Gy

### Initial primary radiation therapy and the salvage SBRT treatment plan criteria

The initial primary radiation therapy plan was generated as previously described [11]. In short, the planning target volume (PTV) was extended 0 to 3 mm from the clinical target volume (CTV). The dose prescription was 70 Gy in 35 fractions for the gross tumor and enlarged lymph nodes, 63 Gy in 35 fractions for the bilateral upper neck, and 56 Gy in 35 fractions for the low-risk region. Simultaneously integrated boost (SIB) IMRT was given to all NPC patients. The treatment plans were generated using the Eclipse treatment planning system (ver. 8.60, Varian Medical Systems, Palo Alto, CA, USA).

For salvage SBRT, the CTV was defined as the locally recurrent nasopharyngeal tumor. The planning target volumes (PTV) were extended 0–2 mm from the corresponding CTVs. The prescription dose and constraints for OARs were based on the initial radiation treatment, radiobiologically adjusted tolerance dose and physician’s ultimate decision. The median prescription dose of the 10 enrolled patients was 36.75 Gy in 5 fractions. A minimum of 95% of the prescription dose was assumed to cover 95% of the PTV. The priority of the treatment planning was sparing of OAR following by target coverage. The details of the planning objectives for the target and OARs are listed in Table 2.

### RapidArc and HyperArc treatment plans

**Fig. 1:**
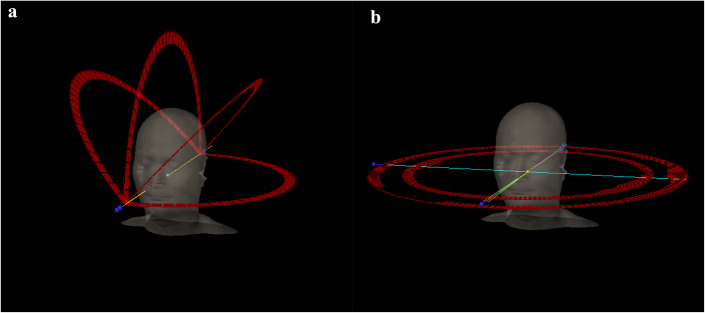
Beam arrangements and arc trajectories of HyperArc (**a**) and RapidArc (**b**). **a** One partial coplanar arc, 0 degrees; three noncoplanar arcs, 45, 90, and 315 degrees; **b** two coplanar arcs

Computed tomography data sets and target volume/normal organ contours from the 10 enrolled patients were transferred to the Eclipse treatment planning system (ver. 15.5, Varian Medical Systems). The virtual Encompass (QFix, Avondale, PA, USA) mask was added only for the HA plans. The corresponding HA and RA plans were then generated according to patient-specific target dose prescription and OAR constraints. 6 MV flattening filter-free photon beams were used, with 1400 MU/min dose rate from a Varian TrueBeam (Varian Medical Systems) linear accelerator equipped with 120-leaf high-definition MLC (with a dynamic beam aperture and a spatial resolution of 2.5 mm leaf width × 32 pairs at the center, 5 mm width × 28 pairs in the peripheral leaves, and maximum static field size 40 cm × 22 cm). In the HA plan, the isocenter was positioned automatically at the center of the selected target structures. The collimator angle and field size were designed to minimize OAR dosages. In addition, the arc fields were also automatically arranged: one full or half coplanar arc with a couch rotation of 0° and up to three partial noncoplanar arcs with couch rotations of 315°, 45°, and 90° (or 270°) [21, 22], respectively (Fig. 1).

In the RA plan, the same isocenter (at the center of the selected target) was set as the HA plan. These two-arc technique (counterclockwise rotation from 179° to 181° and clockwise rotation backwards) was applied for all RA treatment plans (Fig. 1). For all HA and RA plans, the optimization and dose calculation were done per the Photon Optimizer and Anisotropic Analytical Algorithm (ver. 15.5.1, Varian Medical Systems). Jaw tracking optimization was applied to the HA and RA plans. The normal tissue objective optimizers were SRS NTO (ver. 15.5.1, Varian Medical Systems) for HA and automatic NTO (ver. 15.5.1, Varian Medical Systems) for RA.

### Cumulative dose to organs at risk

The dosimetric data of the two treatment courses, including the initial radiation therapy and the salvage SBRT (RA and HA treatment plans, including CT images, structure sets, and radiation doses), were exported from Eclipse to Velocity (ver. 3.2.1, Varian Medical Systems) for each enrolled patient, except one patient whose initial treatment plan had been generated by the Pinnacle treatment planning system (Philips Radiation Oncology Systems, Fitchburg, WI, USA). The cumulative doses to the OARs from the two treatment courses were calculated by the deformable multipass registration method using Velocity [24]. The BED of the OARs were also calculated by the following formula [25, 26]: BED = n × d (1 + d/α/β), where n stands for number of treatment fractions, d is dose per fraction in Gy, and α/β ratio is assumed to be 3 Gy for all OARs. Thus, the relation between BED and EQD2 for the OARs in this study is given as: BED = EQD2 (1 + 2/3) = 1.66 × EQD2, with unit of Gy_3_.

### Plan evaluation statistics

#### Plan evaluation criteria

The parameters used to evaluate the quality of the planned dose distributions for both the HA and RA plans were target coverage, sparing of OARs, and dosimetric parameters mainly recommended by the report from the AAPM Task Group 101 [27].

#### Dosimetric parameters and treatment efficiency

The treatment plans were evaluated by comparing the dosimetric parameters derived from the DVHs for target coverage and sparing of OARs. D2 stands for the dose to 2% of the CTV or PTV, and D98 stands for the dose to 98% of the CTV or PTV, each describing the maximum and minimum dose for the target volumes, respectively. The conformity index (CI), as previously described [28, 29], was defined as follows:

(prescription isodose volume × target volume)/ (volume of the target covered by the prescription isodose volume) ^2^.

The homogeneity index (HI) was determined as the ratio of the highest dose received by 5% of the PTV to the lowest dose received by 95% of the PTV [27].

The intermediate dose spillage was determined as the ratio of the volume of 50% of the prescription isodose curve to the PTV. High dose spillage was calculated as the ratio of the volume outside the PTV that received > 105% of the prescription dose to the PTV volume (V [V105% - PTV] / [PTV]).

Additionally, we calculated the gradient radius as the difference between the equivalent sphere radii of the volume of 50% of the prescription isodose curve and the prescription isodose volume [30]. Monitor units (MUs) and the delivery time were used to assess treatment efficiency.

### Statistical analysis

The dosimetric endpoints of the target volumes, OARs, CIs, HI, intermediate and high dose spillage, the gradient radius, MUs, and the physical dose and BED to OARs were analyzed using the Wilcoxon signed rank test (SPSS, ver. 19, IBM, NY, USA). All tests were 2-tailed, and a *p*-value < 0 .05 was considered statistically significant.

## Results

### Target volume coverage

**Fig. 2:**
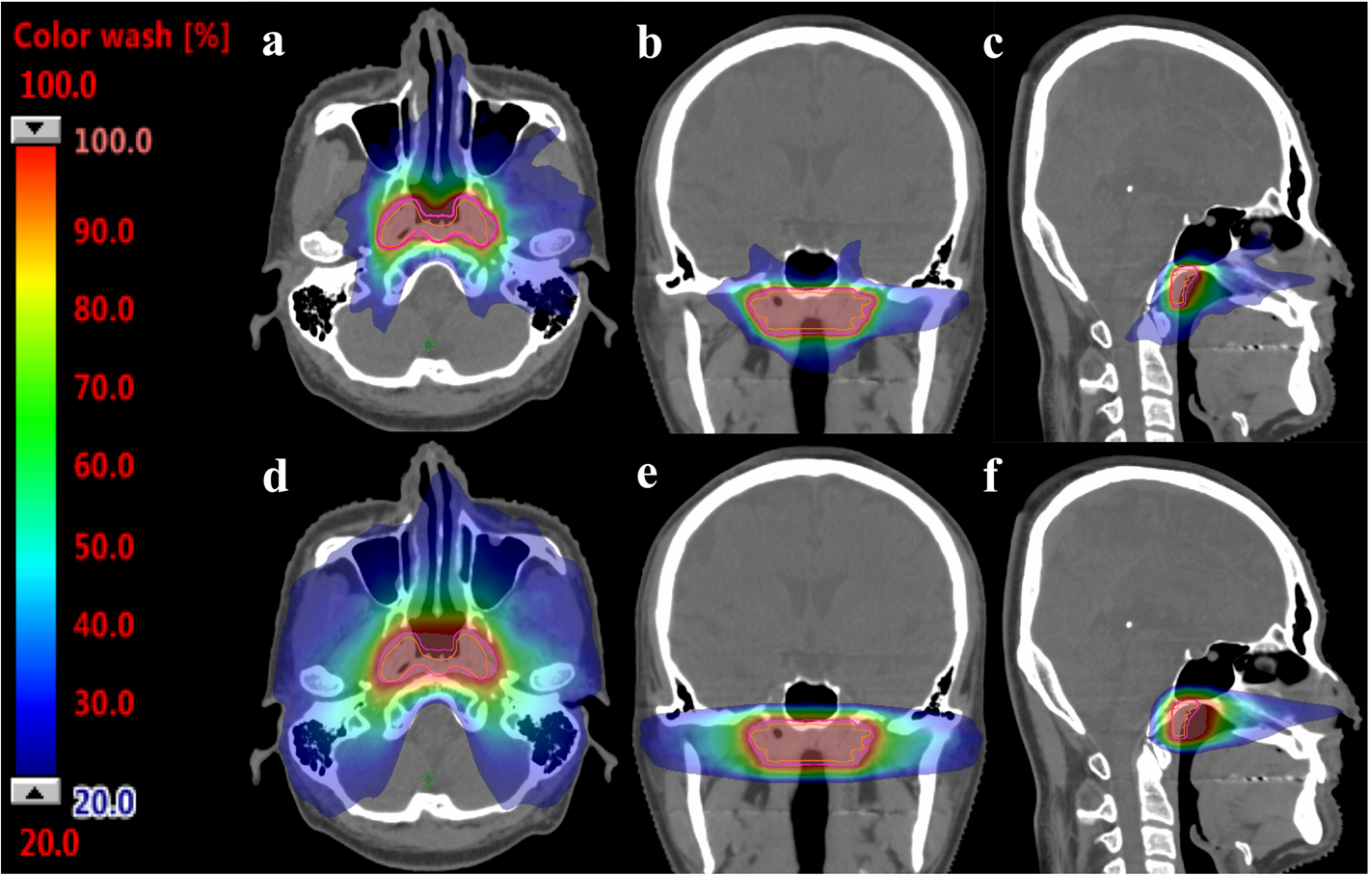
Isodose curves for the applied HyperArc and RapidArc plans. Clinical target volume: orange; planning target volume: pink. **a-c** The HyperArc plan. **d-f** The RapidArc plan. Color wash dose level, from 20% of the prescription dose to 100% of the prescription dose

A detailed comparison of the dosimetric parameters of HA and RA plans for the recurrent NPC patients treated with salvage SBRT is shown in Table 3. HA plans had higher CTV and PTV coverages than RA plans, but both differences were not significant (CTV: 97.71% vs. 95.72%, *p* = 0.214; PTV: 94.12% vs. 92.05%, *p* = 0.333). HA plans did exhibit higher mean dose to targets than RA plans that achieved statistical significance (CTV: 38.09 Gy vs. 37.53 Gy, *p* = 0.007; PTV: 37.75 Gy vs. 37.02 Gy, *p* = 0.005). The isodose curves in the respective HA and RA plans for a patient case are presented in Fig. 2. As one can see, the 50% isodose line conformed better around the target region in the HA plan compared to that in the RA plan, while the 20% isodose line in the RA plan was mostly spread out in the lateral and anterior directions.

**Table 3:** Comparison of dosimetric parameters between salvage HyperArc and RapidArc for recurrent nasopharyngeal cancer patients

Parameters	HyperArc Mean (SEM)	RapidArc Mean (SEM)	*p* value
**Target**			
CTV coverage (%)	97.71 (1.02)	95.72 (1.89)	0.214
D2 (Gy)	38.32 (1.38)	38.42 (1.04)	0.508
D98 (Gy)	35.73 (1.31)	31.85 (2.72)	0.445
Mean dose	38.09 (1.05)	37.53 (1.01)	0.007*
PTV coverage (%)	94.12 (1.48)	92.05 (2.24)	0.333
D2 (Gy)	38.74 (0.93)	38.45 (1.03)	0.139
D98 (Gy)	31.81 (2.71)	30.37 (2.94)	0.767
Mean dose	37.75 (1.07)	37.02 (1.08)	0.005*
**Organs at risk**			
Spinal cord (Gy, Dmax)	3.35 (1.08)	3.32 (1.08)	0.878
Spinal cord (Gy, mean)	1.84 (0.66)	1.30 (0.39)	0.203
Brainstem (Gy, Dmax)	4.26 (1.41)	4.45 (1.38)	0.203
Brainstem (Gy, mean)	0.68 (0.19)	0.64 (0.20)	0.445
Optic nerve_right (Gy, Dmax)	2.22 (0.65)	1.62 (0.89)	0.059
Optic nerve_left (Gy, Dmax)	1.82 (0.59)	1.00 (0.27)	0.005*
Chiasma (Gy, Dmax)	2.46 (0.80)	1.00 (0.12)	0.013*
Eye_right (Gy, Dmax)	1.22 (0.47)	1.79 (1.02)	0.959
Eye_left (Gy, Dmax)	1.48 (0.55)	1.68 (1.03)	0.203
Lens_right (Gy, Dmax)	0.46 (0.08)	0.48 (0.17)	0.093
Lens_left (Gy, Dmax)	0.50 (0.10)	0.41 (0.10)	0.028
Brain (Gy, Dmax)	24.40 (2.79)	26.46 (2.54)	0.017*
Brain (Gy, mean)	1.50 (0.20)	0.76 (0.13)	0.009*
Brain V12 (c.c.)	3.79 (1.04)	8.54 (2.39)	0.022*
Temporal lobe (Gy, Dmax)	20.34 (2.82)	20.31 (3.22)	0.878
Temporal lobe (mean)	4.28 (0.44)	2.13 (1.11)	0.074
Body sum >100Gy (c.c.)	48.66 (9.63)	64.85 (11.16)	0.008*
**Dose distribution metrics**			
Conformity index	1.22 (0.04)	1.42 (0.05)	0.007*
Homogeneity index	1.11 (0.02)	1.17 (0.05)	0.241
High dose spillage	0.03 (0.01)	0.08 (0.04)	0.445
Intermediate dose spillage	3.79 (0.40)	6.05 (0.55)	0.005*
Gradient radius (cm)	0.83 (0.04)	1.21 (0.05)	0.005*
Monitor units	21,390 (1561)	13,467 (1816)	0.037*

Abbreviations: *CTV* Clinical target volume, *PTV* Planning target volume, *D2* The radiation dose to 2% of the CTV or PTV, *D98* The radiation dose to 98% of the CTV or PTV, *Body sum >100Gy* The body volume that received accumulated doses more than 100 Gy from the primary and salvage sessions. SEM, standard error of the mean; *, statistically significant, *p* < 0.05

### Sparing of organs at risk

**Fig. 3:**
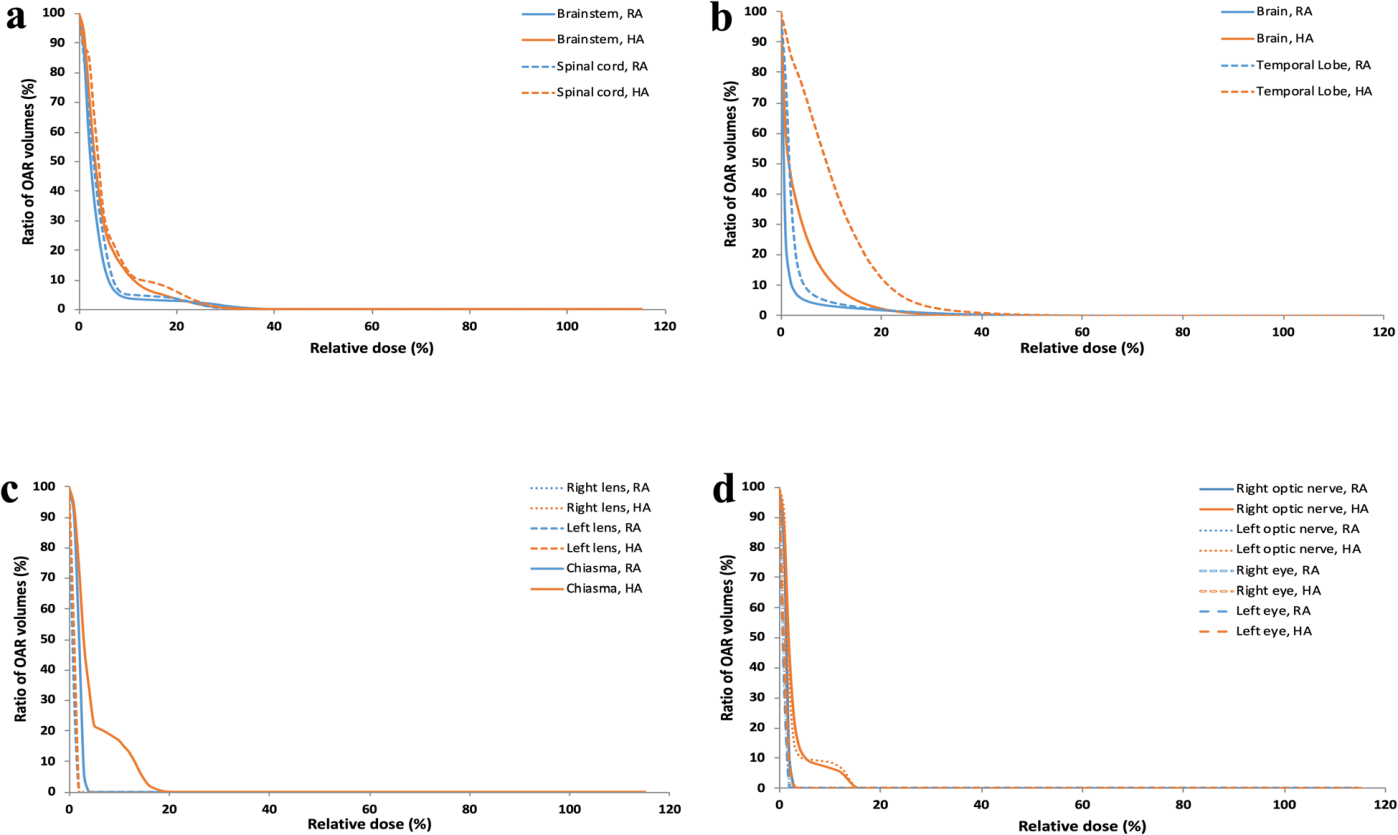
Comparison of the mean dose-volume histograms for the HyperArc (HA) and RapidArc (RA) techniques. **a-d** Organs at risk (OAR); X axis, relative dose, the percentage of the prescription dose; Y axis, ratio of OAR volumes, the volume percentage of the OAR

Table 3 also includes dose-volume parameters for sparing of OARs in salvage HA vs. RA treatments. All requirements for critical organ-dose constraints for re-irradiation alone were satisfied. The maximum and mean doses to brainstem and spinal cord as generated in HA plans were not significantly different from those generated in RA plans. Significant differences were observed between HA and RA plans in the maximal doses to the optic apparatus (specifically optic nerve_left, *p* = 0.005; optic chiasm, *p* = 0.013) and in the mean brain dose (*p* = 0.009). Using RA technique, the mean maximal doses to the optic nerves and optic chiasm and mean brain dose were reduced by 1 to 1.5 Gy. With HA technique, the volume of brain receiving 12 Gy and more (V12) was decreased by 44% (HA vs. RA: 3.79 c.c. vs. 8.54 c.c., *p* = 0.022), while the mean maximal dose to the brain was significantly less than the RA technique (HA vs. RA: 24.40 Gy vs. 26.46 Gy, *p* = 0.017). Figure 3 shows the difference in mean dose-volume histograms between HA and RA plans for OAR sparing.

### Comparison of dosimetric parameters

#### Conformity and homogeneity indices

**Fig. 4:**
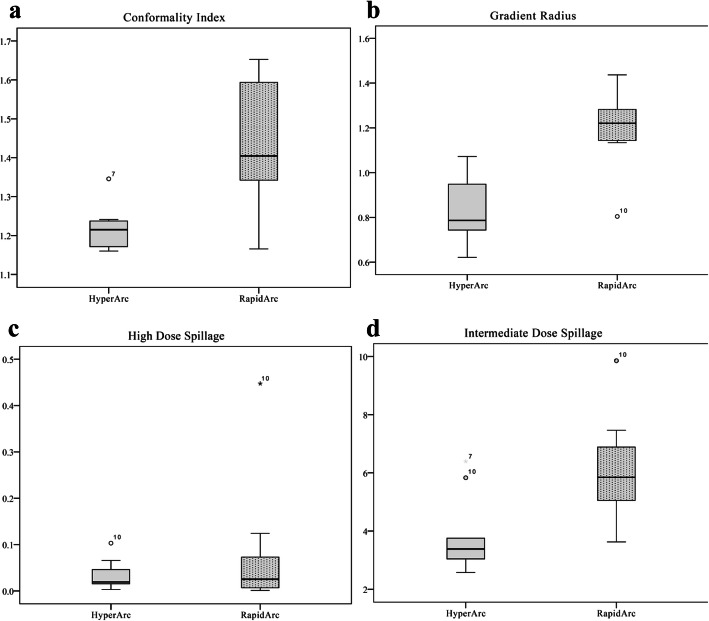
Boxplots of the dosimetric parameters for the HyperArc and RapidArc plans. **a** conformity index; **b** gradient radius; **c** high dose spillage; **d** intermediate dose spillage. Boxes, median value and upper and lower quartiles; Whiskers, maximum and minimum values within 1.5 interquartile range; Dots, outliers

Figure 4 shows the box plot of the dosimetric parameters with regard to CI, gradient radius, high dose spillage, and intermediate dose spillage for both salvage treatment techniques. The mean CI and HI are shown in Table 3. The HA plans showed a higher degree of conformity (CI: HA, 1.22 vs. RA, 1.42, *p* = 0.007) while achieving slightly more homogenous dose distribution than RA plans without statistical significance (HI: HA, 1.11 vs. RA, 1.17, *p* = 0.241).

#### Dose gradient

Intermediate dose spillage was determined by the ratio of the volume of 50% of the prescription isodose curve to the PTV. For such measure of dose gradient, HA showed significantly faster dose fall-offs than RA technique did (HA vs. RA: 3.79 vs. 6.05, *p* = 0.005). Similar high dose spillages were observed in HA than in the RA plans (HA vs. RA: 0.03 vs. 0.08, *p* = 0.445). The mean gradient radius in HA plans (0.83 cm) was significantly shorter than that in RA plans (1.21 cm).

#### Cumulative biological doses to organs at risk

For biologically meaningful comparisons, Table 4 provides the individual BED values to the OARs for the initial primary treatment and either salvage SBRT, as well as the cumulative BED for the entire sequential treatment course with either salvage technique (P + HA vs. P + RA). For salvage re-irradiation alone, there was significantly less BED to the optic apparatus in RA plans (BED to optic nerve_left: HA > RA, *p* = 0.005; BED to chiasm: HA > RA, *p* = 0.013), but a significant reduction of the brain BED in HA plans (BED to brain, HA < RA, *p* = 0.028). For the combined treatment, the cumulative BEDs to the optic apparatus (optic chiasm, optic nerves, and left eye) in the P + RA plans were indeed significantly less than those in the P + HA plans, while the cumulative BED to the brain in the P + HA plans was significantly less than that in the P + RA plans (*p* = 0.038).

**Table 4:** Biologically effective dose to the OARs from initial primary treatment, and salvage HyperArc or RapidArc SBRT for recurrent nasopharyngeal cancer patients

Parameters	Primary treatment Mean (SEM)	Salvage HA Mean (SEM)	Salvage RA Mean (SEM)	p value	Cumulative dose (P + HA) Mean (SEM)	Cumulative dose (P + RA) Mean (SEM)	*p* value
**Organs at risk (Gy**_**3**_**)**							
Spinal cord	58.14 (1.91)	4.80 (1.99)	4.62 (2.02)	0.878	63.33 (2.77)	61.57 (1.92)	0.173
Brainstem	67.73 (2.39)	6.65 (3.02)	6.91 (3.08)	0.203	72.18 (2.15)	72.38 (1.83)	0.260
Brain	114.83 (2.39)	68.78 (12.23)	77.03 (11.77)	0.028*	176.85 (12.20)	185.94 (11.62)	0.038*
Temporal lobe	115.66 (2.09)	52.69 (11.99)	54.05 (12.43)	0.799	159.90 (9.94)	161.24 (9.44)	0.767
Chiasma	13.10 (3.12)	3.25 (1.24)	1.08 (0.14)	0.013*	16.56 (3.25)	14.10 (3.08)	0.008*
Optic nerve_right	27.86 (6.47)	2.80 (0.94)	2.27 (1.50)	0.074	30.08 (6.30)	28.64 (6.46)	0.008*
Optic nerve_left	29.12 (6.86)	2.25 (0.87)	1.12 (0.34)	0.005*	31.16 (6.63)	29.90 (6.85)	0.008*
Eye_right	22.87 (7.20)	1.45 (0.66)	2.62 (1.80)	0.959	23.67 (7.18)	23.70 (7.28)	0.515
Eye_left	20.08 (6.37)	1.81 (0.80)	2.51 (1.82)	0.203	21.12 (6.41)	20.77 (6.38)	0.038*

Abbreviations: *Gy*_*3*_ Unit of BED with α/β ratio of 3 Gy, *SEM* Standard error of the mean; *, statistically significant, p < 0.05; *HA* HyperArc, *RA* RapidArc, *P + HA* Primary treatment plan+ HyperArc treatment plan, *P + RA* Primary treatment plan+ RapidArc treatment plan

#### Treatment efficiency

Across all fractions of the salvage SBRT treatment, HA plans generated an average of 21,390 MUs, as compared to 13,467 MUs generated by RA plans (*p* = 0.0037). The median estimated delivery time from dry runs for RA plans was 3:26 min per fraction (range: 2:31 min. to 5:52 min.) and that for HA plans was 5:17 min per fraction (range: 4:59 min. to 6:40 min.). The median difference in the time of delivery was less than 2 min (111 s), without considering patient alignment and imaging acquisition.

## Discussion

There are only a few reports that have mentioned the use of modern VMAT technique for recurrent nasopharyngeal cancer in the salvage SBRT setting [31]. Here, we report the dosimetric results of 10 recurrent NPC patients by creating salvage SBRT treatment plans and demonstrate the feasibility of such salvage re-irradiation approaches by comparing the noncoplanar VMAT (HA) with the coplanar VMAT (RA) technique. Our study was prompted by the lack of data concerning the treatment of recurrent NPC using SBRT with the goal of minimizing the cumulative radiation dose to pertinent OARs.

In general, RA is able to produce a high-quality treatment plan and achieve fast delivery of SRS or SBRT [18, 32]. The RA plans in this study were generated with Eclipse treatment plan system (Varian Medical System, Palo Alto, CA) for a commercial LINAC system (TrueBeam, Varian Medical System, Palo Alto, CA) equipped with high-definition MLCs to deliver radiotherapy beams in a stereotactic, isocentric coplanar fashion. Such VMAT technique has been reported to result in a tightly conformed homogeneous dose distribution over intended target volumes while reducing low-dose areas in the periphery [32, 33]. Here, we demonstrated that RA plans consistently reduced the radiation dose to multiple OARs simultaneously with good coverage of the targets.

There are also VMAT techniques other than the RA approach. The HA technique allows for a partial or full coplanar arc and up to 3 noncoplanar arcs in an isocentric fashion. In the current study, we demonstrated that the most distinguishing advantage of HA over RA is being able to consistently achieve better CI, gradient radius and intermediate dose spillage. As a result of the improved CI and dose fall-off with the HA technique in comparison with RA, a higher mean target dose was consistently achieved in HA plans without compromising the CTV or PTV coverage. Therefore, HA can produce a very high-quality treatment plan with excellent dosimetric parameters.

One of the concerns for salvage re-irradiation approach is the potential toxicity to critical normal structures from the accumulative radiation dose in time. Few studies have performed detailed dosimetric analyses to analyze the added toxicities due to overlapped dose coverage from the initial primary radiotherapy and salvage SBRT. Again, at any anatomic site of interest the combined biologic effects of sequentially separated radiation treatment courses of distinct fractionation schemes cannot be inferred from the simple summation of total radiation doses deposited. We have thus employed the concept of BED (or EQD2) to guide our salvage SBRT planning (Table 2) and provided the dosimetric comparisons between RA & HA techniques separately as well as in combination with the initial primary treatment (Table 4).

The cumulative dosage, either in terms of physical or biologically effective dose, to OARs only showed minor differences between HA and RA plans. In certain OARs, such as the optic apparatus and temporal lobe, the cumulative dose from the primary radiotherapy and salvage SBRT plans were significantly different between the RA and HA plans, but the difference was of a small magnitude (1–4 Gy, or 0.03–2.15Gy_3_). The difference in OAR doses between HA and RA was seen to be a result of the different arc trajectories. Enabling the spread-out of radiation dose is a characteristic of noncoplanar beam delivery. At the very least, more entrance and exit doses from the noncoplanar arcs would lead to wider low-dose regions. The radiobiological significance of OARs embedded within such a low-dose bath remains unknown. The current algorithm of HA technique is very effective in controlling high and intermediate dose spreading and fall-off while maintaining conformity. For example, the brain V12, an indicator of brain necrosis, was significantly smaller with HA than with RA technique (HA vs. RA: 3.79 c.c. vs. 8.54 c.c., *p* = 0.022). The maximal dose of the brain in HA was also significantly less than in RA plans (HA vs. RA: 24.40Gy vs. 26.46Gy, *p* = 0.017).

Neurologic organs are relatively easy to identify and their treatment induced side effects are easier to evaluate. However, toxicities to soft tissues such as trismus or muscle fibrosis, along with the dose-toxicity relationships, are more difficult to evaluate but could nonetheless truly impact patient’s quality of life. It has been reported that patients who were retreated with a cumulative external beam dose of > 100 Gy had a high incidence of severe complications [34]. In our study, HA significantly decreased the body volume that received > 100 Gy as compared to RA technique.

There are still some limitations to this current study. First, the 10 enrolled patients had diverse treatment plan criteria for the initial primary radiation treatments before the subsequent salvage SBRT treatments. This limitation reflected the reality of heterogeneous clinical presentation of recurrent NPC cases. While we were unable to optimize the initial treatment plans that had been delivered, we could maximize the quality of the salvage treatment plans. Both HA and RA techniques showed excellent target dose coverages while sparing OARs. Second, in the current study, we only summed the BEDs of the initial primary radiotherapy and salvage SBRT treatment, without considering the factor of possible normal tissue repairs during the time in-between which might also influence the degree of ultimate toxicity expression. For neurological tissues, for example, the tolerance to cumulative dose may be enhanced with the increase in the time interval between the initial treatment and re-irradiation [35, 36]. It may be challenging to analyze these kinetic parameters since the efficiency of such latent repair often remains imprecisely known for various OARs of interest. In theory, the very fact that BED is “additive” which we try to illustrate in the current study also reflects the feasibility of incorporating such normal tissue protective effect – but only if future research could provide more robust quantitative data. It would be analogous to what has been done by past investigators in accounting for the effect of “accelerated repopulation” for acute responding tissues [37]. Regardless, by ignoring the plausible “protective” effect for normal tissue repair during a prolonged time interval, the dose constraints as listed in Table 2 would represent even more conservative estimates independent from the chosen reirradiation technique of either RA or HA approach. Third, there have not been clinically significant outcomes observed with either HA or RA technique so far. It remains unknown what degree of late toxicity might be caused by small increments of total radiation doses or BEDs for certain OARs. With increasing understanding and skills about how to prevent severe treatment-related toxicities [38, 39], the high-quality VMAT treatment planning might continue to help improve the therapeutic ratio of salvage SBRT treatment for recurrent NPC cases.

## Conclusions

The novel dosimetric distributions in conformality, homogeneity and low dose spillage make the HA technique an attractive SBRT option for the salvage treatment of recurrent NPC. It should be noted that certain OARs under the arc trajectory of HA (e.g., optic apparatus) that had received substantial amount of dose during the initial course of primary radiation treatment might accumulate more radiation dose than the same OARs would with RA technique. Further clinical studies using HA for recurrent NPC would be necessary to confirm the therapeutic benefits and the toxicity profiles.

## Data Availability

Not applicable.

## Abbreviations

SBRT: Stereotactic body radiation therapy
RA: RapidArc
HA: HyperArc
OARs: Organs at risk
NPC: Nasopharyngeal cancer
VMAT: Volumetric modulated arc radiotherapy
MLC: Multileaf collimator
PTV: Planning target volume
CTV: Clinical target volume
EQ D2: Equivalent dose to 2 Gy per fraction
BED: Biologically effective dose
CI: Conformity index
HI: Homogeneity index
MUs: Monitor units
SRS: Stereotactic radiosurgery
